# Prevalence and genetic diversity of coronaviruses, astroviruses and paramyxoviruses in wild birds in southeastern Kazakhstan

**DOI:** 10.1016/j.heliyon.2022.e11324

**Published:** 2022-10-31

**Authors:** Andrey V. Zhigailov, Elina R. Maltseva, Yuliya V. Perfilyeva, Yekaterina O. Ostapchuk, Dinara A. Naizabayeva, Zhanna A. Berdygulova, Saltanat A. Kuatbekova, Anna S. Nizkorodova, Akzhigit Mashzhan, Andrey E. Gavrilov, Almat Zh. Abayev, Ilyas A. Akhmetollayev, Seidigapbar M. Mamadaliyev, Yuriy A. Skiba

**Affiliations:** a Almaty Branch of the National Center for Biotechnology, Almaty, Kazakhstan; b M.A. Aitkhozhin Institute of Molecular Biology and Biochemistry, Almaty, Kazakhstan; c Tethys Scientific Society, Almaty, Kazakhstan; d Al-Farabi Kazakh National University, Almaty, Kazakhstan; e Institute of Zoology, Almaty, Kazakhstan

**Keywords:** Astroviruses, Coronaviruses, Paramyxoviruses, *RdRp* gene, Wild birds

## Abstract

Wild birds are natural reservoirs of many emerging viruses, including some zoonoses. Considering that the territory of Kazakhstan is crossed by several bird migration routes, it is important to know pathogenic viruses circulating in migratory birds in this region. Therefore, the aim of this study was to identify the host range, diversity and spatial distribution of avian paramyxoviruses, coronaviruses, and astroviruses in free-ranging wild birds in the southeastern region of Kazakhstan. For this purpose, we collected tracheal and cloacal swabs from 242 wild birds belonging to 51 species and screened them using conventional PCR assays. Overall, 4.1% (10/242) and 2.9% (7/242) of all examined birds tested positive for coronaviruses and astroviruses, respectively. Coronaviruses were found in the orders Pelecaniformes (30%; 3/10), Charadriiformes (30%; 3/10), Columbiformes (20%; 2/10), Anseriformes (10%; 1/10), and Passeriformes (10%; 1/10). All detected strains belonged to the genus *Gammacoronavirus*. Astroviruses were detected in birds representing the orders Passeriformes (57%; 4/7), Coraciiformes (14%; 1/7), Charadriiformes (14%; 1/7), and Columbiformes (14%; 1/7). Paramyxoviruses were observed in only two birds (0.8%; 2/242). Both strains were closely related to the species APMV-22, which had not been previously detected in Kazakhstan. Phylogenetic analysis of the partial *RdRp* gene sequences of the virus strains revealed three different clades of astroviruses, two clades of coronaviruses, and one clade of paramyxoviruses. The results of this study provide valuable information on the diversity and spatial distribution of paramyxoviruses, coronaviruses, and astroviruses in wild birds in southeastern Kazakhstan and highlight the importance of further thorough monitoring of wild birds in this region.

## Highlights

• First study on CoVs and AstroVs in wild birds in Kazakhstan.
• APMVs, CoVs and AstroVs are confirmed by RT-PCR and partial *RdRp* gene sequencing.
• The CoVs prevalence is higher in aquatic birds as compared to terrestrial species.
• The obtained CoV strains belong to the genus *Gammacoronavirus*
• Strains closely related to APMV-22 not previously detected in Kazakhstan are shown.

## 1 Introduction

Three of the eight major global migration routes for terrestrial and waterbirds cross the territory of Kazakhstan, including the West Asian/East African, Central Asian and Black Sea/Mediterranean flyways (BirdLife International, 2022). In addition, southeastern Kazakhstan is partially crossed by the fourth East Asian/Australasian flyway. Several wader species belonging to the order Charadriiformes stop here on their way from Siberia and the Russian Far East to Indonesia and northern Australia (Gavrin et al., 1962). After a long flight through the mountain ridges of the Pamir, Tibet, and the Tien Shan the migratory birds need to stop and replenish their strength for an onward flight. Wetlands of the southeastern region of Kazakhstan, surrounded by the Tien Shan mountain ridge in the south and the Dzhungarian Alatau and Altai ridges in the east, are attractive gathering places for a wide range of avian species.

Wild birds are important natural reservoirs and potential dispersers of a variety of highly pathogenic viruses. Due to their flocking behavior and ability to fly long distances, they have the potential to efficiently spread emerging pathogens among themselves, poultry, livestock, and humans (Lau et al., 2018). In particular, wild birds have been implicated in the spread of paramyxoviruses (APMVs), coronaviruses (CoVs), and astroviruses (AstroVs), the causative agents of significant diseases of avian and mammalian species including humans.

According to the International Committee on Taxonomy of Viruses (ICTV), APMVs are placed under the subfamily *Avulavirinae* of the family *Paramyxoviridae.* and represented by three genera, including *Orthoavulavirus*, *Metaavulavirus* and *Paraavulaviru*s (Rima et al., 2019). To date, 22 species of APMVs (APMV-1 to APMV-22) that cause diseases with various clinical presentations in a wide variety of avian species have been identified (Rao et al., 2020). Coronaviruses belong to the family *Coronaviridae*, subfamily *Orthocoronavirinae*. They cause respiratory, hepatic, enteric, and neurological disorders in mammals and birds (Lau et al., 2018; Łukaszuk and Stenzel, 2020). Alpha-CoVs and beta-CoVs are found in mammals, whereas gamma-CoVs and delta-CoVs are mainly found in birds (Woo et al., 2012). Avian astroviruses are members of the genus *Avastrovirus* of the family *Astroviridae*. According to ICTV, three species of the genus *Avastrovirus* including *Avastrovirus 1, Avastrovirus 2*, and *Avastrovirus 3* have been described (Todd et al., 2009). These viruses cause gastrointestinal disorders in poultry and are frequently detected in birds with enteritis as well as in clinically healthy ones (Cortez et al., 2017).

While surveillance of avian influenza viruses is conducted effectively in Kazakhstan (Lewis et al., 2021), surveillance of APMVs is sporadic (Bogoyavlenskiy et al., 2009; Karamendin et al., 2016, 2017), and to our knowledge, there are no data on the prevalence and genetic diversity of avian CoVs and AstroVs circulating in Kazakhstan. Meanwhile, the diseases caused by these viruses can lead to substantial economic losses to commercial poultry. Therefore, the aim of this study was to identify the host range, diversity, and spatial distribution of APMVs, CoVs and AstroVs in free-ranging wild birds in southeastern Kazakhstan.

## 2 Materials and methods

### 2.1 Ethics statement

This study was approved by the local ethical committee of the Institute of Genetics and Physiology of the Committee of Science of the Ministry of Education and Science of the Republic of Kazakhstan (approval # 2019-12-No2). All experimental procedures were performed in accordance with the International Guiding Principles for Biomedical Research Involving Animals.

### 2.2 Sample collection

**Figure 1:**
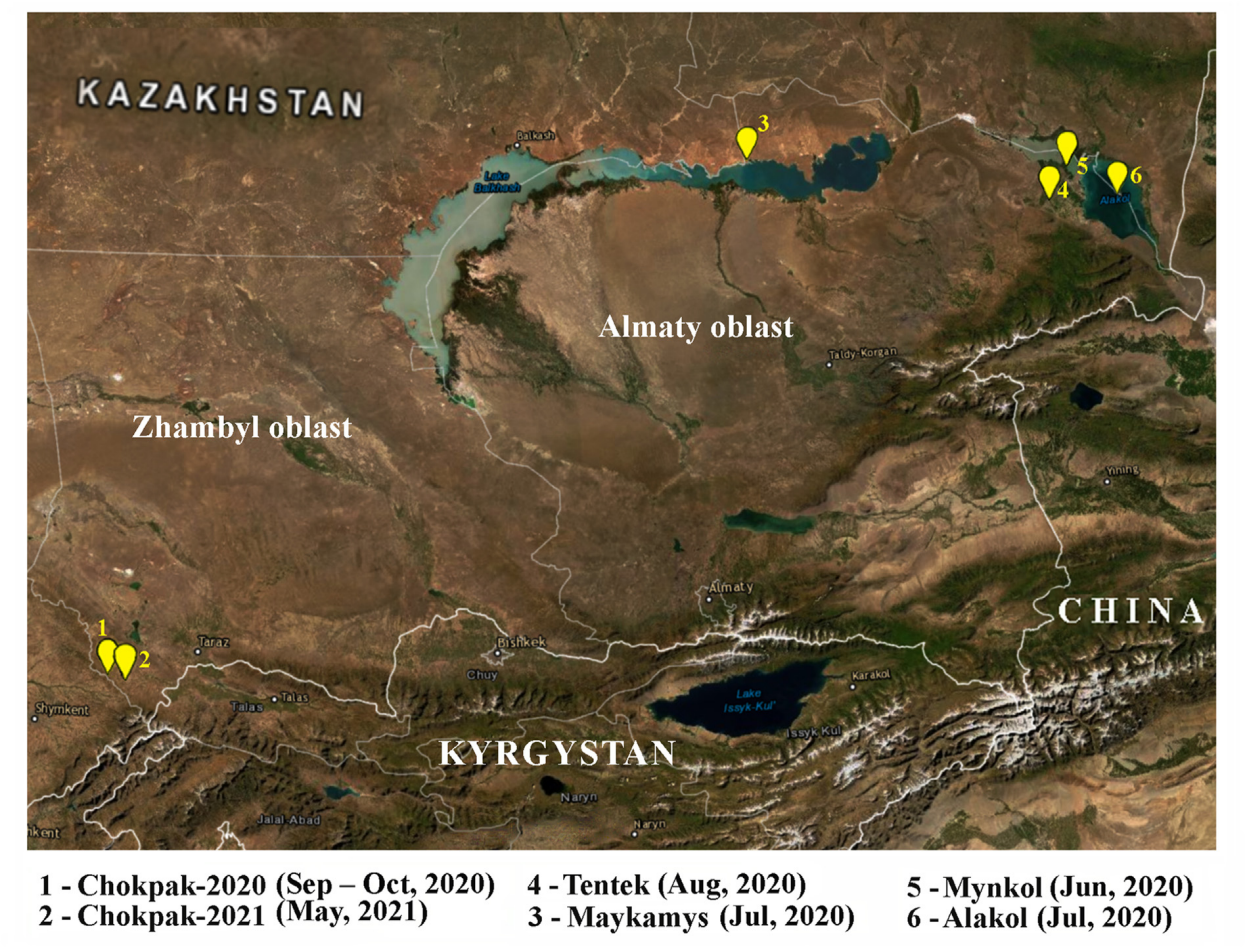
Sites of sample collection in southeastern Kazakhstan. Map was generated using the ArcGIS Earth version 1.14.0.3321.

Tracheal and cloacal swabs were collected from individual birds using sterile swabs, then placed into viral transport medium (Multitrans Collection and Transportation System, Starplex) and transferred to the laboratory in liquid nitrogen. Selection of sampling sites was based on the location of major migratory habitats. Samples from terrestrial birds were collected mainly at the ornithological station in the Chokpak Pass located between the Talas Alatau and Karatau mountain ranges in Zhambyl oblast. Samples were collected in 2020 and 2021 at the Chokpak-2020 (42°33′50″N, 70°37′04″E) and Chokpak-2021 (42°30′56″N, 70°39′51″E) sites, respectively. Samples from aquatic birds were collected in 2020 at four collection sites: Maykamys village on the northern coast of Lake Balkhash (46°39′02″N, 77°34′22″E), Tentek River (46°14′20″N 80°59′46″E), Mynkol Lakes (46°25′42″N, 81°13′37″E) and Big Alakol Island (46°11′32″N, 81°46′01″E) in Almaty oblast (Figure 1). After sampling, all captured birds were released back to the source habitat.

A total of 242 migratory and non-migratory birds representing 12 orders, 26 families, and 51 species were sampled at six locations in southeastern Kazakhstan (Table 1). Aquatic and terrestrial birds accounted for 27.7% (67/242) and 72.3% (175/242), respectively. Migratory birds outweighed resident birds in both the number of individuals collected (81%; 196/242 and 19%; 46/242, respectively) and across tested species (86%; 44/51 and 14%; 7/51, respectively). Most birds captured belonged to the order Passeriformes (44.2%; 107/242). Detailed information regarding bird species is presented in Table 2. Only 34.3% (83/242) birds were sampled in 2021, while all other birds (65.7%; 159/242) were captured and sampled in 2020.

**Table 1:** Summary of data on birds sampled in southeastern Kazakhstan during the period 2020–2021.

Avian order	Chokpak-2020	Chokpak-2021	Maykamys	Tentek	Mynkol	Alakol	Total (%)
Falconiformes	24	2					26 (10.7%)
Strigiformes	5	3					8 (3.3%)
Caprimulgiformes		6					6 (2.5%)
Columbiformes	13	1					14 (5.8%)
Coraciiformes	7	6					13 (5.4%)
Charadriiformes			40	8		1	49 (20.2%)
Passeriformes	41	64		2			107 (44.2%)
Galliformes		1					1 (0.4%)
Anseriformes				1		4	5 (2.1%)
Pelecaniformes					11		11 (4.6%)
Ciconiiformes				1			1 (0.4%)
Podicipediformes						1	1 (0.4%)
Total (%):	90 (37.2%)	83 (34.3%)	40 (16.5%)	12 (5.0%)	11 (4.5%)	6 (2.5%)	242

**Table 2:** Data on RT-PCR-positive samples obtained from birds in southeastern Kazakhstan.

Order	Family	Species	No of birds	No (%) PCR-positive samples		
				CoV	APMV	AstroV
Falconiformes	Accipitridae	*Pernis ptilorhynchus*	1			
		*Milvus migrans*	7			
		*Circus cyaneus*	2			
		*Accipiter gentilis*	2			
		*Accipiter nisus*	5			
		*Buteo buteo*	6			
	Falconidae	*Falco subbuteo*	2			
		*Falco naumanni*	1			
Strigiformes	Strigidae	*Asio otus*	2			
		*Asio flammeus*	1			
		*Otus scops*	5			
Columbiformes	Columbidae	*Columba palumbus*	2		1 (50%)	
		*Columba oenas*	4	1 (25%)	1 (25%)	
		*Columba eversmanni*	1			
		*Columba livia*	2	1 (50%)		1 (50%)
		*Streptopelia orientalis*	5			
Coraciiformes	Meropidae	*Merops apiaster*	12			1 (8%)
	Coraciidae	*Coracias garrulus*	1			
Charadriiformes	Laridae	*Larus ichthyaetus*	1	1 (100%)		
		*Hydroprogne caspia*	40			1 (3%)
		*Larus ridibundus*	2	1 (50%)		
	Scolopacidae	*Tringa totanus*	4	1 (25%)		
	Charadriidae	*Vanellus vanellus*	2			
Passeriformes	Hirundinidae	*Riparia diluta*	11	1 (9%)		
		*Hirundo rustica*	13			1 (8%)
		*Hirundo daurica*	1			
	Sturnidae	*Sturnus roseus*	3			
		*Sturnus vulgaris*	1			
	Corvidae	*Corvus monedula*	6			2 (33 %)
		*Corvus frugilegus*	9			1 (11%)
		*Corvus corone*	4			
		*Corvus cornix*	4			
	Turdidae	*Turdus atrogularis*	1			
		*Oenanthe pleschanka*	4			
		*Luscinia luscinia*	1			
	Fringillidae	*Fringilla coelebs*	2			
		*Fringilla montifringilla*	2			
	Emberizidae	*Emberiza leucocephalos*	1			
		*Emberiza calandra*	3			
	Muscicapidae	*Muscicapa striata*	2			
	Laniidae	*Lanius minor*	3			
	Passeridae	*Passer hispaniolensis*	34			
	Sylviidae	*Acrocephalus dumetorum*	1			
	Paridae	*Parus bokharensis*	1			
Galliformes	Phasianidae	*Perdix perdix*	1			
Caprimulgiformes	Caprimulgidae	*Caprimulgus europaeus*	6			
Pelecaniformes	Pelecanidae	*Pelecanus crispus*	11	3 (27%)		
Ciconiiformes	Ardeidae	*Nycticorax nycticorax*	1			
Anseriformes	Anatidae	*Tadorna ferruginea*	4	1 (25%)		
		*Anas platyrhynchos*	1			
Podicipediformes	Podicipedidae	*Podiceps cristatus*	1			
**Total:**			**242**	**10 (4.1%)**	**2 (0.8%)**	**7 (2.9%)**

### 2.3 RNA extraction and cDNA synthesis

The swabs were stored at −80°С. Viral RNA was extracted using an RNeasy Mini Kit (Qiagen). RNA was eluted in 40 μl RNase-free water and used immediately for RT-PCR or stored at −80°С until further analysis. Complementary DNA (cDNA) was synthesized using a SuperScript IV Reverse Transcriptase Kit (Thermo Fisher scientific) according to the manufacturer’s instructions using random hexamer primers.

### 2.4 PCR assay

All samples were screened for APMVs, CoVs and AstroVs using a conventional RT-PCR assay. The primers used in the study are listed in Table 3.

**Table 3:** Primers for PCR screening assays.

Pathogen	Name	Sequence	Genome position∗	Target	T_a_ [°C]	Product size, bp	Reference
**Primers for detection and (or) molecular characterization of paramyxoviruses**							
*Paramyxovirinae*	PAR-F1	(5′)GARGGNYDRTGYCARAARHTDTGGAC	10543–10568^a^		50	650–656	(Tong et al., 2008), modified
*Paramyxovirinae* (seminested PCR assay)	PAR-R PAR-F2	(5′)GCTGAAGTTACNGGHTCHCCDATRTTBC	11177–11204^a^	*L* gene (RdRp locus)			
		(5′)GTTGCTTCAATGGTTCARGGNGAYAA	10621–10646^a^			573–579	
*Avulavirus*, *Rubulavirus*	PAR-Avu-F4	(5′)AGYRTNTTYHTNAARGAYAARGCAAT	9784–9809^a^		53	836–926	This study
	PAR-Avu-R4	(5′)ATTRCYTGATTRTCWCCYTGNACCAT	10630–10655^a^				
**Primers for detection, typing and(or) molecular characterization of coronaviruses**							
*Coronaviridae*	pan-CoV-outF	(5′)CCAARTTYTAYGGHGGBTGG	14117–14137^b^	*Orf1a,b* gene (*RdRp* locus)	53	670–673	(Xiu et al., 2020), modified
	pan-CoV-outR	(5′)TGTTGHGARCARAAYTCATGDGG	14767–14789^b^				
*Coronaviridae* (nested PCR assay)	CoV-RdRp-F2	(5′)GGTTGGGACTATCCTAAGTGTGA	14188–14210^b^		53	436–440	(Chu et al., 2011)
	CoV-RdRp-R2	(5′)CCATCATCAGATAGAATCATCAT	14605–14627^b^				
*Coronaviridae* (nested PCR assay)	CoV-RdRp-F3	(5′)GGKTGGGAYTAYCCKAARTG	14188–14207^b^		53	599–602	(Chu et al., 2011)
	CoV-RdRp-F3	(5′)TGYTGTSWRCARAAYTCRTG	14770–14789^b^				
*Gammacoronavirus*	CoV-Hel-GAM-F	(5′)GTGYDGGTAGYGAAAAYGTTGATGAT	15428–15453^b^	*Orf1a,b* gene (*Hel* locus)	54	1123	This study
	CoV-Hel-GAM-R	(5′)AARCACTSRCGTGATTCTGGGT	16529–16550^b^				
*Deltacoronavirus*	CoV-Hel-DEL-F	(5′)ATCAACAAYTACATTTGTAGTGTTGA	13772–13797^c^		54	1436–1451	This study
	CoV-Hel-DEL-R	(5′)GMTGCTTTNRCATTCATAGCATT	15185–15207^c^				
*Coronaviridae* (nested PCR assay)	CoV-Hel-Pan-F	(5′)TTYGCDGCWGARACNVTHARRGC	15535–15557^b^		50	461–475	This study
	CoV-Hel-Pan-R	(5′)TTRCCDSTRCCDGGDGGDCC	15982–16001^b^				
**Primers for detection and molecular characterization of astroviruses**							
*Astroviridae*	AstroV-UNI-F	(5′)TTGAYTGGACNMGHTWTGATGGYAC	4141–4165^d^	*Pol* gene (*RdRp* locus)	50	417–447	(Todd et al., 2009), modified
	AstroV-UNI-R	(5′)CWGGYTTVACCCACATNCCAAA	4563–4584^d^				


∗ Primer positions refer to the sequences of:^a^*Newcastle disease virus* (APMV-1) strain JS10, (GenBank: HQ008337);^b^*Gammacoronavirus* strain IBV M41 (GenBank: DQ834384);^c^*Wigeon coronavirus HKU20* strain HKU20-9243 (GenBank: JQ065048);^d^*Duck astrovirus* strain SL2 (GenBank: KF753805).

For CoV detection, the initial RT-PCR run was performed with the outer primers ‘pan-CoV-outF’ and ‘pan-CoV-outR’ (Xiu et al., 2020), which target the RNA-dependent RNA polymerase (*RdRp*) region of the *ORF1a,b* gene (Table 3). For the second PCR reaction we used two pairs of inner primers ‘CoV-RdRp-F2’/‘CoV-RdRp-R2’ and ‘CoV-RdRp-F3’/‘CoV-RdRp-R3’ (Chu et al., 2011) (Table 3). In addition, for CoV detection and further PCR-typing of CoV-positive samples, two nested PCR assays were conducted. Two pairs of outer primers ‘CoV-Hel-GAM-F’ and ‘CoV-Hel-GAM-R’, which target the helicase (*Hel*) locus of the gamma-CoVs *Orf1a,b* gene, as well as ‘CoV-Hel-DEL-F’ and ‘CoV-Hel-DEL-R’, which target the *Hel* locus of the delta-CoVs *Orf1a,b* gene, were used. For both nested PCR assays, the inner primers ‘CoV-Hel-Pan-F’ and ‘CoV-Hel-Pan-R’ were used yielding an amplification product of 461–475 bp.

For APMV detection, a seminested PCR assay was performed using pan-PMV primers (Table 3) targeting a conserved fragment of the *L-*gene encoding RdRp (Tong et al., 2008). Because the pan-PMV primers do not detect all APMV species, we used additional degenerate primers ‘PAR-Avu-F4’ and ‘PAR-Avu-R4’ (Table 3), which amplify a 670–674 bp fragment of the *L-*gene of avulaviruses and rubulaviruses.

Universal degenerate primers (Table 3) targeting a conserved *RdRp* region of the *ORF1b* gene were utilized to detect the presence of AstroVs (Todd et al., 2009).

Hot-Taq DNA polymerase (SibEnzyme) was used for virus detection. Amplification for sequencing was performed using High-Fidelity Taq Polymerase (Thermo Fisher scientific) according to the manufacturer’s instructions. The conditions for PCR amplification were as follows: 94 °C for 10 min, 94 °C for 40 s, 50–54 °C (T_a_ values for each primer pair are indicated in Table 3) for 30 s, 72 °C for 1 min, followed by 5 min at 72 °C; 40 cycles were performed. PCR products were analyzed by 1.5% agarose gel electrophoresis and visualized under UV light.

### 2.5 Sequencing and phylogenetic analysis

Elution of DNA fragments from agarose gels was performed using a QIAquick gel extraction kit (Qiagen). The purified DNA fragments were then sequenced in both directions using a BigDye® Terminator v3.1 Cycle Sequencing Kit (Applied Biosystems) and ABI 3500XL genetic analyzer (Applied Biosystems). Sequences were aligned using the MUSCLE algorithm. An unrooted phylogenetic tree was generated with the Maximum Likelihood Algorithm using the MEGA X software. The bootstrap method with 1000 replicates was used to evaluate the reliability of the tree topologies.

### 2.6 Statistical analysis

Statistical analysis was performed using EpiInfo v. 7.2.2.2 software (CDC). The exact Clopper-Pearson method based on the beta distribution was used to calculate the 95% confidence interval (CI). Pearson’s chi-square test was used to determine a statistically significant difference between groups. Differences were considered statistically significant at *p* < 0.05.

## 3 Results

All collected samples were examined for viruses belonging to three families, *Paramyxoviridae*, *Coronaviridae* and *Astroviridae* using RT–PCR assays.

### 3.1 Detection and genetic characterization of CoVs

Using the nested PCR targeting the *RdRp* locus we identified eight positive samples for CoVs (3.3%; 95% CI: 1.4–6.4%). The nested PCR targeting the *Hel* locus detected 7/242 or 2.9% (95% CI: 1.2–5.9%) of samples positive for CoVs. By combining the results of both PCR assays, 10/242 or 4.1% (95% CI: 2.0–7.5%) of all samples tested positive for CoVs in at least one PCR assay.

Ten CoV strains were found in eight avian species from five orders (Table 2). The detection rates of CoVs were 27% for Pelecaniformes (3/11; 95% CI = 6.0–61.0%), 20% for Anseriformes (1/5; 95% CI = 0.1–71.6%), 14% for Columbiformes (2/14; 95% CI = 1.8–42.8%), 6% for Charadriiformes (3/49; 95% CI = 1.3–16.9%), and 0.9% for Passeriformes (1/107; 95% CI = 0.01–5.1%). All detected CoVs were found to be gamma-CoVs. Delta-CoVs were not detected. RNA samples positive for CoV were found at four of the six locations tested including Chokpak-2020, Mynkol, Alakol and Tentek. The prevalence of CoVs was higher in aquatic birds (10%; 7/67; 95% CI = 4.3–20.4%) as compared to terrestrial birds (1.7%; 3/175; 95% CI = 0.4–4.9%; χ^2^ = 7.25; *p* = 0.0071).

**Figure 2:**
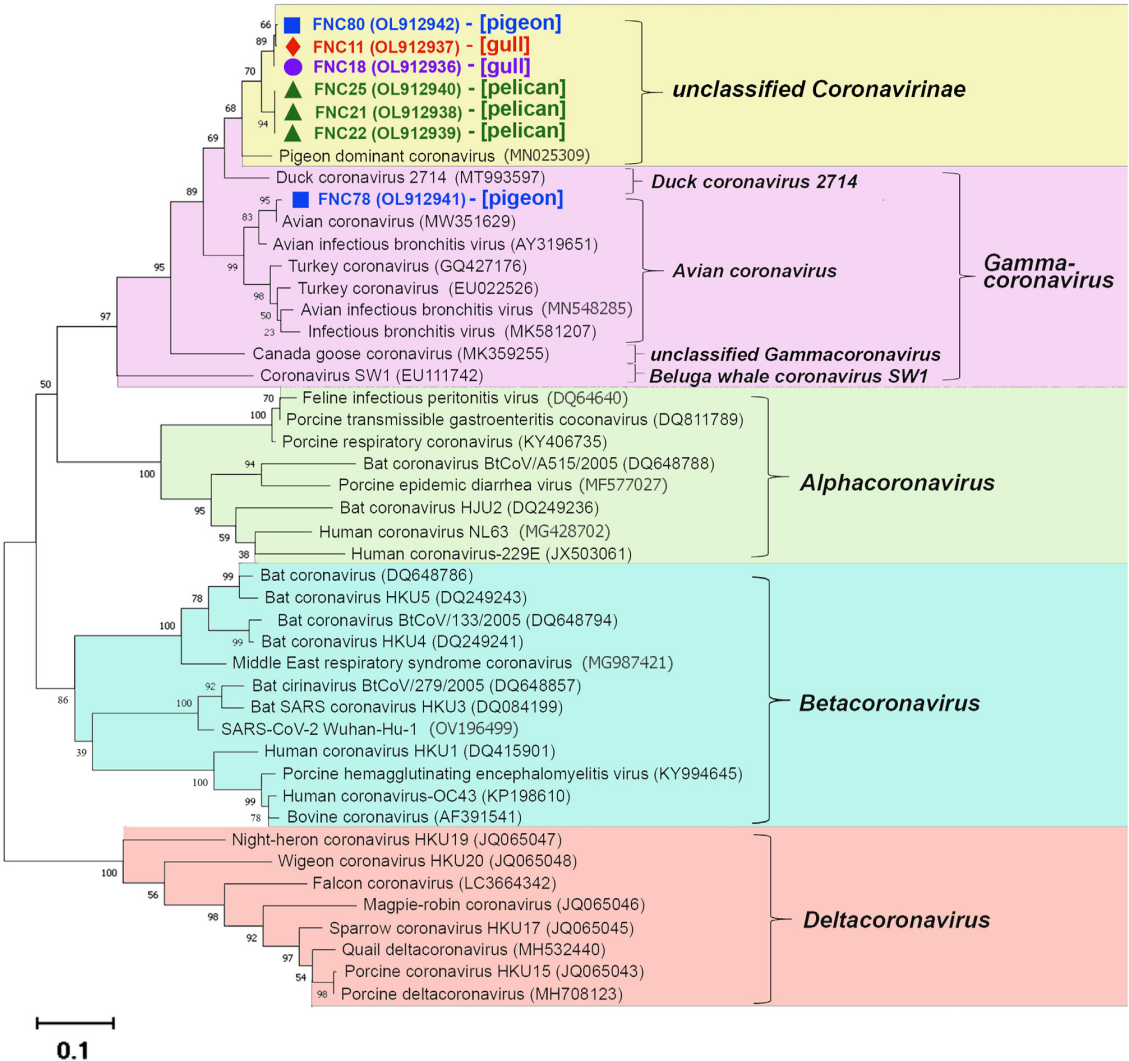
Phylogenetic tree of avian CoVs based on partial sequences (461–475 bp) of the *Hel* locus of the *Orf1a,b* gene. Sites of CoV sample collection are indicated as follows: Chokpak-2020 – square (■), Mynkol – triangle (▲), Tentek – rhombus (♦), Alakol – cycle (●). The scale bar corresponds to 0.1 substitutions per nucleotide position. GenBank accession numbers are indicated in parentheses. The hosts of CoV strains identified in this study are indicated in square brackets.

Blast analysis of the sequences of the amplified CoV *Orf1b Hel* locus fragments confirmed that the detected CoVs belonged to the genus *Gammacoronavirus* (data not shown). Unfortunately, three of the ten CoV sequences obtained were of insufficient quality for further phylogenetic analysis. The phylogenetic tree for seven strains analyzed is shown in Figure 2. One of the strains (GenBank: OL912941) was closely related to avian infectious bronchitis virus strains. Six strains clustered into a distinct clade and shared 89–92% nucleotide sequence similarity with the pigeon-dominant CoV. Nine of ten CoV-positive samples were found in migratory avian species.

Phylogenetic analysis revealed no pronounced clustering of strains based on geographic location or host species. Two gamma-CoV strains (GenBank: OL912941 and OL912942) that shared only 80% nucleotide identity were detected in pigeons captured at Chokpak-2020. At the same time, six genetically close gamma-CoV strains (GenBank: OL912936 to OL912940 and OL912942) with more than 95% nucleotide identity were identified in both aquatic (pelicans, gulls, sandpipers) and terrestrial (pigeons) bird species that are evolutionarily distinct.

### 3.2 Detection and genetic characterization of APMVs

Only two samples were positive for APMV RNA (0.8%; 95% CI = 0.1–3.0%) as detected by both PCR assays. Positive samples were collected from pigeons at the Chokpak-2020 location. The overall prevalence of APMVs in Columbiformes was shown to be 14% (2/14; 95% CI = 1.8–42.8%).

**Figure 3:**
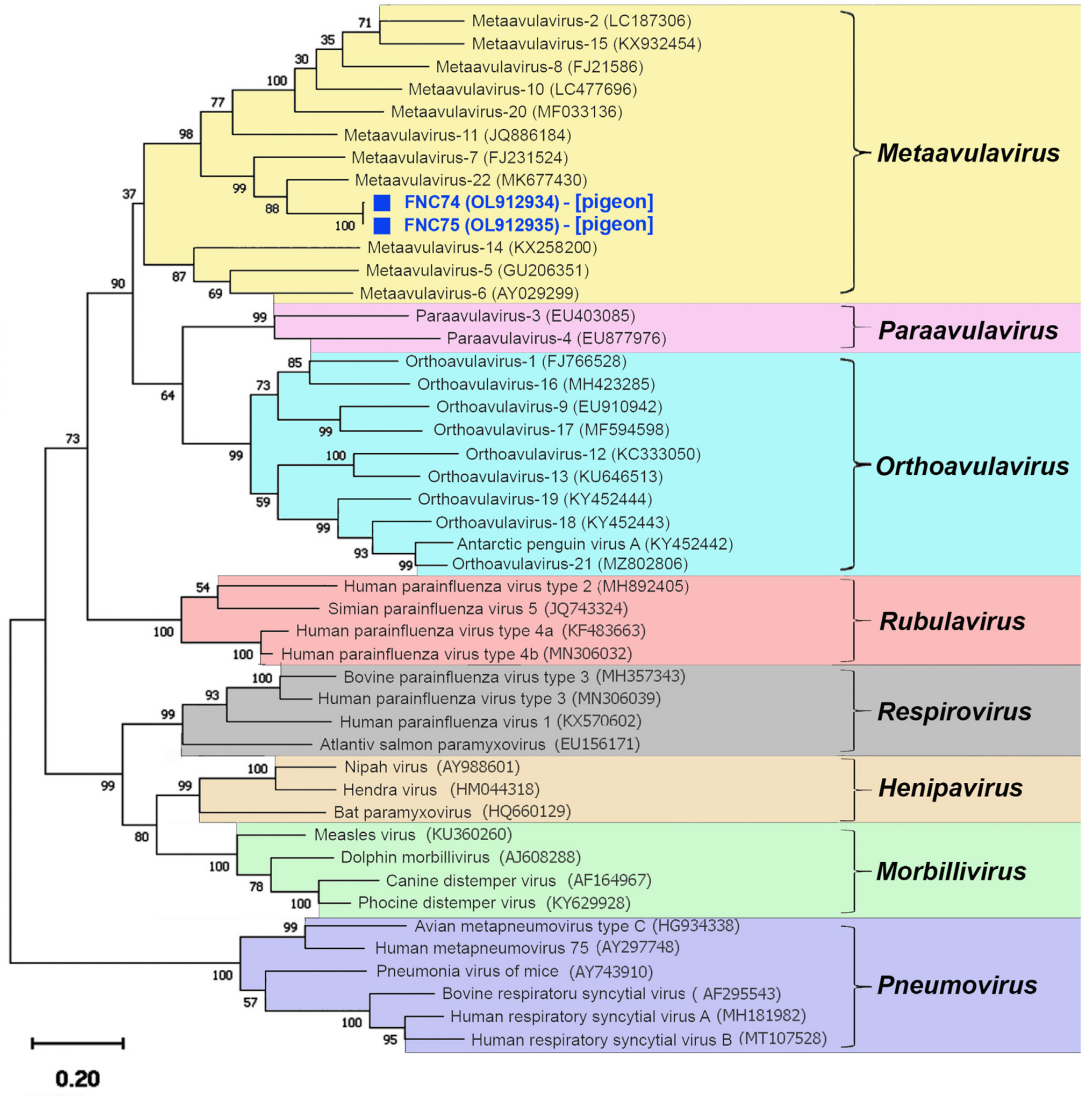
Phylogenetic tree of APMV species based on *RdRp*-gene partial sequences (649–686 bp). Squares (■) indicate the APMV strains obtained in this study (Chokpak-2020 location). The scale bar corresponds to 0.2 substitutions per nucleotide position. GenBank accession numbers are indicated in parentheses. The hosts of APMV strains identified in this study are indicated in square brackets.

The partial *L-*gene sequences of the obtained APMV strains had a nucleotide identity of 75.6% with APMV-21 (GenBank: MK677430) and 72.5% with APMV-7 (GenBank: FJ231524) (Figure 3). Although the partial *L-*gene sequences of these two strains were almost identical, they were collected from pigeons of two different species (*C. palumbus* and *C. oenas*).

### 3.3 Detection and genetic characterization of AstroVs

Screening for AstroVs demonstrated an overall prevalence of 2.9% (7/242; 95% CI = 1.2–5.9%). Seven AstroV strains were detected in six species (Table 2). These viruses were more frequently detected in the order Coraciiformes (8%; 1/13; 95% CI = 0.2–36.0%), followed by the orders Columbiformes (7%; 1/14; 95% CI = 0.2–33.9%), Passeriformes (3.7%; 4/107; 95% CI = 1.0–9.3%), and Charadriiformes (2%; 1/49; 95% CI = 0.1–10.9%). Most of the AstroV-positive birds belonged to the family Corvidae of the order Passeriformes (Table 2). One AstroV-positive sample was collected at the Maykamys location, while six AstroV-positive samples were collected at the Chokpak-2020 location. We found no difference in the AstroV prevalence between terrestrial (3.4%, 6/175; 95% CI = 1.3–7.3%) and aquatic birds (2%, 1/67; 95% CI = 0.01–8.0%; χ^2^ = 0.14; *p* = 0.7073). Six of seven AstroV-positive samples were found in migratory avian species.

**Figure 4:**
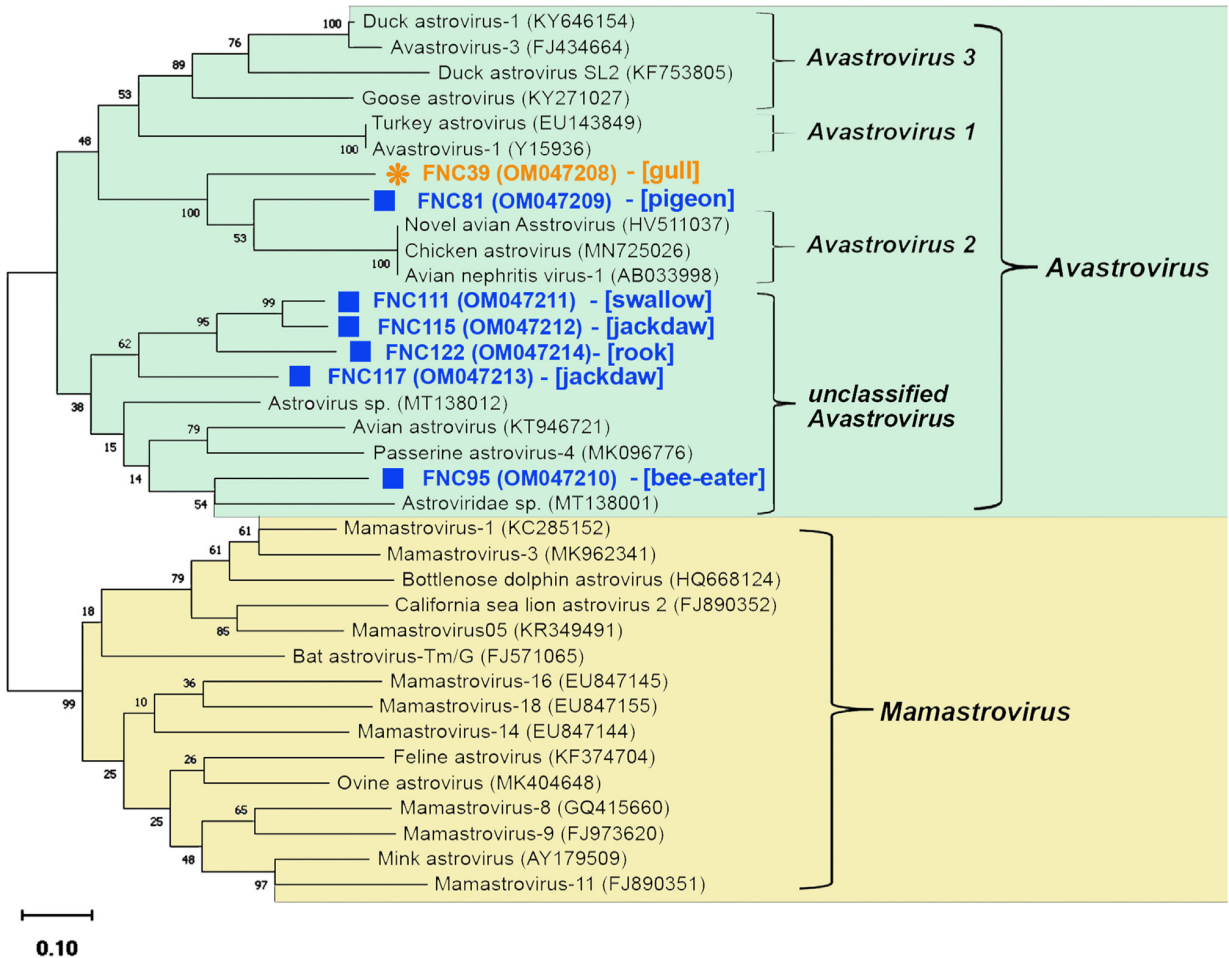
Phylogenetic tree of AstroVs based on *RdRp-gene* partial sequences (417–447 bp). Sites of AstroV sample collection are indicated as follows: Chokpak-2020 – square (■), Maykamys – asterisk (∗). The scale bar corresponds to 0.1 substitutions per nucleotide position. GenBank accession numbers are indicated in parentheses. The hosts of AstroV strains identified in this study are indicated in square brackets.

Phylogenetic analysis showed that all seven AstroV strains belonged to the genus *Avastrovirus* (Figure 4). The analysis revealed genetic diversity among AstroV strains originating from the same geographical area (Chokpak Pass) and clustering based on host species (families Corvidae, Columbidae, Laridae and Meropidae) (Figure 4, Table 2).

## 4 Discussion

The current study is the first to describe the presence of CoVs and AstroVs in wild migratory and resident birds in southeastern Kazakhstan. The study also describes the prevalence of APMVs circulating in wild birds in this region, Across 51 avian species tested, APMVs were found in only two pigeon species captured at migratory bird resting and feeding areas representing an overall APMV prevalence of 0.8%. The obtained data are consistent with a previous study analyzing samples collected in Kazakhstan between 2002 and 2013, which showed that the prevalence of APMVs in wild birds ranged between 0.15% and 2.73% (Karamendin et al., 2016). These data suggest that APMVs spread in the studied bird species with relatively low efficiency.

Gamma-CoVs were detected in wild birds more frequently with an overall prevalence of 4.1%. Despite the use of a screening method capable of detecting both gamma- and delta-CoVs (Chu et al., 2011), we were unable to detect the latter in our study. Consistent with a number of previously reported studies, the frequency of gamma-CoVs was significantly higher in waterfowl as compared to terrestrial birds, indicating their role as important natural reservoirs or intermediate hosts of gamma-CoVs in the region (Chamings et al., 2018; Chu et al., 2011; Miłek and Blicharz-Domańska, 2018; Paim et al., 2019; Wille et al., 2015). Gamma-CoVs were more frequently detected in Pelecaniformes followed by Anseriformes.

The prevalence of AstroVs was 2.9%, which can be considered relatively low. AstroVs were detected in apparently healthy birds representing six different avian species. The data confirm a relatively broad avian host range of AstroVs described previously (Pantin-Jackwood et al., 2012; Fernández-Correa et al., 2019; Chu et al., 2012). Interesting enough, there was a notable difference in the proportion of AstroV-positive samples among the studied areas. Six of seven PCR-positive samples were detected at Chokpak-2020. Although the obtained results can be explained by a considerably lower number of birds sampled at other locations in 2020 as compared to the Chokpak location, they may also indicate a higher importance of some flyways for the AstroV transmission.

We observed seasonal fluctuations in the prevalence of CoVs, AstroVs and APMVs in wild birds sampled at the same location (Chokpak Pass). All 83 samples collected in the spring season at Chokpak Pass (Chokpak-2021 location) were negative for all viruses examined, whereas we found CoV-, AstroV- and APMV-positive samples among the 90 samples collected in the fall season at the same location (Chokpak-2020 location). Similar seasonality is frequently described for avian influenza A virus (Wille et al., 2015; Xu et al., 2016). The observed fluctuations could be related to a number of factors, including ambient temperature and humidity, which may affect the period of virus survival in bird secretions and aerosols, as well as the direction of migration. Infected birds are less likely to successfully complete the long flights over the ridges of Pamir and Tien Shan during spring migration from wintering to breeding grounds. Infected birds are also more likely to die during wintering due to a general weakening of the body. Alternatively, the difference could be explained by a different composition of avian species sampled in the spring and fall seasons. In particular, there were no representatives of the order Columbiformes in the spring collection, which showed a relatively high virus prevalence in the fall collection.

Although the number of samples for phylogenetic analysis was limited, we identified three different AstroV clades, two CoV clades, and one APMV clade. We showed that at least one strain of gamma-CoV is quite widespread in southeastern Kazakhstan. It was detected in several bird species (pelicans, gulls, waders, pigeons) in both Almaty and Zhambyl oblasts of Kazakhstan suggesting that it is capable of wide transmission between different bird species. This CoV clade is closely related to the pigeon-dominant CoV, which is common in neighboring China (Zhuang et al., 2020).

We then showed that the two APMV strains detected in pigeons shared over 70% nucleotide identity with APMV-21 and APMV-7. The distribution of APMV-1 (Karamendin et al., 2016), APMV-2 (Saiatov et al., 1992), APMV-4, APMV-6, APMV-8 (Bogoyavlenskiy et al., 2009) APMV-15 (Karamendin et al., 2017) and APMV-20 (Karamendin et al., 2017) in Kazakhstan have been previously described. Neither APMV-7 nor APMV-22 strains have yet been identified in Kazakhstan. APMV-7 is mainly found in North America (Xiao et al., 2012) and it is highly unlikely to enter Kazakhstan with migratory birds. APMV-21 has been detected in Taiwan (Liu et al., 2019). APMV-22 strains may have entered Kazakhstan with migratory waders travelling along the East Asian/Australasian flyway. It is also possible that the APMV strains identified in this study belong to a new, as yet uncharacterized species.

Four of the seven detected AstroV strains (GenBank: OM047211 to OM047214) clustered into a clade that may be a new species of the genus *Astrovirus*. All four samples were collected from birds belonging to the order Passeriformes. These genetically related strains originated from different geographical regions, the south of Zhambyl oblast and the Alakol-Balkhash region of Almaty oblast. The absence of serious geographical obstacles may facilitate virus transmission between these two regions of Kazakhstan. One AstroV strain (GenBank: OM047210) was obtained from Merops apiaster and showed the highest identity to Astroviridae sp. (GenBank: MT138001). The results of the phylogenetic analysis also support the possibility that the other two AstroV strains collected from Hydroprogne caspia (GenBank: OM047208) and Columba livia (GenBank: OM047209) are distinct species, as the evolutionary distance between these strains is as much as 35%.

This study has several limitations. The main limitation is the small number of samples. For future work, samples should be collected throughout the year to evaluate seasonality. Another limitation is that the phylogenetic analysis was performed with very short genome fragments. Complete genome sequencing is needed to verify whether the identified divergent AMPV and AstroV strains belong to new species.

## 5 Conclusion

This is the first study describing the prevalence and genetic diversity of avian CoVs and AstroVs in southeastern Kazakhstan. Phylogenetic analysis of partial viral genome sequences revealed three major AstroV clades and two CoV clades. In addition, we described APMVs that had not previously been detected in Kazakhstan. Further studies are needed to describe the full picture of the ecology of CoVs, AstroVs and APMVs in Kazakhstan.

## Declarations

### Author contribution statement

Conceived and designed the experiments; Performed the experiments; Analyzed and interpreted the data; Contributed reagents, materials, analysis tools or data; Wrote the paper.

Andrey V. Zhigailov: Performed the experiments; Analyzed and interpreted the data; Wrote the paper.

Elina R. Maltseva: Conceived and designed the experiments; Contributed reagents, materials, analysis tools or data.

Yuliya V. Perfilyeva and Yekaterina O. Ostapchuk: Analyzed and interpreted the data; Wrote the paper.

Andrey E. Gavrilov and Seidigapbar M. Mamadaliyev: Conceived and designed the experiments.

Yuriy A. Skiba: Analyzed and interpreted the data.

Zhanna A. Berdygulova, Akzhigit Mashzhan and Almat Zh. Abayev: Performed the experiments.

Dinara A. Naizabayeva, Saltanat A. Kuatbekova, Anna S. Nizkorodova and Ilyas A. Akhmetollayev: Contributed reagents, materials, analysis tools or data.

### Funding statement

Andrey E. Gavrilov, Seidigapbar M. Mamadaliyev and Yuriy A. Skiba were supported by 10.13039/501100004561Ministry of Education and Science of the Republic of Kazakhstan [АР08855831, AP09259102 & AP09259103].

### Data availability statement

Data associated with this study has been deposited at GenBank under the accession number OL912934 to OL912942 (CoVs), OM047208 to OM047214 (AstroVs), OL912934 and OL912935 (APMVs).

### Declaration of interest’s statement

The authors declare no conflict of interest.

### Additional information

No additional information is available for this paper.
